# Populations Are Differentiated in Biological Rhythms without Explicit Elevational Clines in the Plant *Mimulus laciniatus*

**DOI:** 10.1177/0748730420936408

**Published:** 2020-07-06

**Authors:** Päivi H. Leinonen, Matti J. Salmela, Kathleen Greenham, C. Robertson McClung, John H. Willis

**Affiliations:** * Department of Ecology and Genetics, University of Oulu, Oulu, Finland; † Department of Biology, Duke University, Durham, North Carolina, USA; ‡ Natural Resources Institute Finland (Luke), Helsinki, Finland; § Department of Biological Sciences, Dartmouth College, Hanover, New Hampshire, USA

**Keywords:** circadian period, environmental variation, *Erythranthe laciniata*, phenological timing, photoperiod

## Abstract

Environmental variation along an elevational gradient can yield phenotypic differentiation resulting from varying selection pressures on plant traits related to seasonal responses. Thus, genetic clines can evolve in a suite of traits, including the circadian clock, that drives daily cycling in varied traits and that shares its genetic background with adaptation to seasonality. We used populations of annual *Mimulus laciniatus* from different elevations in the Sierra Nevada in California to explore among-population differentiation in the circadian clock, flowering responses to photoperiod, and phenological traits (days to cotyledon emergence, days to flowering, and days to seed ripening) in controlled common-garden conditions. Further, we examined correlations of these traits with environmental variables related to temperature and precipitation. We observed that the circadian period in leaf movement was differentiated among populations sampled within about 100 km, with population means varying by 1.6 h. Significant local genetic variation occurred within 2 populations in which circadian period among families varied by up to 1.8 h. Replicated treatments with variable ecologically relevant photoperiods revealed marked population differentiation in critical day length for flowering that ranged from 11.0 to 14.1 h, corresponding to the time period between late February and mid-May in the wild. Flowering time varied among populations in a 14-h photoperiod. Regardless of this substantial population-level diversity, obvious linear clinality in trait variability across elevations could not be determined based on our genotypic sample; it is possible that more complex spatial patterns of variation arise in complex terrains such as those in the Sierra Nevada. Moreover, we did not find statistically significant bivariate correlations between population means of different traits. Our research contributes to the understanding of genetic variation in the circadian clock and in seasonal responses in natural populations, highlighting the need for more comprehensive investigations on the association between the clock and other adaptive traits in plants.

Phenotypic differentiation along environmental gradients can result from variable selection pressures between habitats (Endler, 1986). Plants predict seasonal changes for timing of important phenological events such as flowering by integrating photoperiod and temperature signals (Amasino, 2010; Andrés and Coupland, 2012). In areas where environmental variation is closely linked with latitude (e.g., northern Europe), corresponding natural variation in seasonal responses occurs for traits such as timing of growth in forest trees (Mikola, 1982) and insect diapause (Tyukmaeva et al., 2011). More complex spatial patterns of adaptation may emerge in mountainous areas, where elevational differences in annual changes in temperature and moisture levels result in environments having different photoperiods at the start of and during the growing season. Because initiation of flowering can have a critical day-length threshold associated with timing of the optimal reproductive season (Garner and Allard, 1920), these elevational gradients can lead to highly localized variation in selective pressures and reciprocal differentiation in flowering time and photoperiodic responses (Kooyers et al., 2015; Levandowska-Sabat et al., 2017; Montesinos-Navarro et al., 2011; Vidigal et al., 2016).

Responses to environmental signals such as photoperiod can involve interactions with the circadian clock—an internal timekeeping oscillator—via coincidence of internal and external signals (Harmer, 2009; Greenham and McClung, 2015; Yanovsky and Kay, 2003). The clock coordinates daily oscillations in various metabolic and physiological functions that are crucial for survival and successful reproduction (Greenham and McClung, 2015; Millar, 2016). Molecular components of the clock are well characterized, and varied traits consistently exhibit circadian period lengths of approximately 24 h in constant conditions (Millar, 2016). Still, ecological determinants of the consequential natural genetic diversity in the clock remain poorly known (Salmela and Weinig, 2019). For example, natural genotypes in common model systems *Arabidopsis thaliana* and *Mimulus guttatus* exhibit free-running circadian periods in leaf movement that vary by more than 5 h across immense latitudinal gradients spanning up to 50° (Greenham et al., 2017; Michael et al., 2003). It has yet to be determined how this diversity relates to variation in specific environmental factors, other adaptive traits (including the manifestation of free-running period length in naturally cycling conditions), and ultimately fitness. In addition, results from studies sampling genotypes on a more regional within-population scope show that the magnitude of genetic diversity even within a few hundred meters can be comparable with that observed across continents (Greenham et al., 2017; Salmela et al., 2016).

Studies on experimental plant progeny suggest genetic associations between the clock, photosynthesis, and growth patterns (Edwards et al., 2011; Rubin et al., 2017; Rubin et al., 2018; Yarkhunova et al., 2018). Furthermore, the complexity of the circadian clock implies that its function comprises both maintenance of day-night cycles and seasonal responses (Troein et al., 2009). In fact, molecular studies and genetic mapping have shown that the circadian clock and regulation of flowering have overlapping genetic backgrounds (Brachi et al., 2010; Lou et al., 2011; Suárez-López et al., 2001). Natural variation in the clock could therefore be correlated with traits that facilitate adaptation to seasonality and with the same environmental factors that shape diversity, for instance, in photoperiodic responses. Although both the clock and phenology are genetically variable locally and globally, their covariation has not been thoroughly explored in natural plant populations (Salmela and Weinig, 2019). In birds, there is evidence that endogenous daily, seasonal reproductive cycles and timing of migration are correlated in situ, for instance, such that early chronotypes tend to initiate nesting earlier (Graham et al., 2017; Rittenhouse et al., 2019). In a population of the plant model *Boechera stricta*, seed families with longer circadian period in their first year tended to flower early and at a larger size after a simulated winter in a common garden (Salmela et al., 2018). Thus, in addition to broad latitudinal gradients, spatially restricted sampling schemes that limit the number of simultaneously varying environmental factors can be used to explore the role of the clock in adaptation (Salmela and Weinig, 2019).

The *Mimulus guttatus* species complex has emerged as a convenient model in ecological genomics (Wu et al., 2008; Zuellig et al., 2014). These species occur in a wide range of habitats where the growing season is largely determined by edaphic conditions (Wu et al., 2010). *Mimulus* harbors natural variation in critical day-length requirement associated with life history and elevation across western North America (Friedman and Willis, 2013), making it an attractive system for studying differentiation in and covariation between the circadian clock and flowering responses. In our study, we used natural populations of *Mimulus laciniatus* A. Gray (Phrymaceae), an annual species that grows on granite outcrops and that is endemic to the California Sierra Nevada (Ferris et al., 2014; Sexton et al., 2011). Growing season on these outcrops is limited by water availability, as soil moisture levels decrease rapidly during summer and effectively end the growing season (Dickman et al., 2019; Ferris and Willis, 2018). The species occurs over an extensive elevational range within its relatively small distribution, where seasonal variation in climate results in the length and timing of the growing season differing markedly across short distances. These factors are likely to impose selection on biological rhythms in *M. laciniatus*. Populations in the region exhibit similar estimates of diversity at molecular markers, moderate genetic differentiation, and evidence for some gene flow between similar environmental settings, even across distinct elevational transects (Sexton et al., 2016). Further, high inbreeding coefficients across the range show that the species principally self-fertilizes (Ferris et al., 2014; Sexton et al., 2011, 2016).

We studied genetic differentiation in the circadian clock, timing of phenological transitions, and flowering responses to photoperiod among populations of *M. laciniatus*. More specifically, we answered the following questions. First, do circadian period length, flowering responses to photoperiod, and timing of phenological transitions (days to cotyledon emergence, flowering, and seed ripening) under permissive day length differ among populations and seed families originating from different elevations in the Sierra Nevada? Second, is variation in these traits associated with local environmental gradients? Based on a previous plant study that quantified among- and within-population variation in circadian period within ~100 km (Salmela et al., 2016), we predicted analogous, potentially elevation-dependent diversity in *M. laciniatus*. We also hypothesized that plants from higher elevations would require longer day lengths to initiate flowering and have delayed phenology as compared with populations from lower elevations (Friedman and Willis, 2013; Kooyers et al., 2015). Finally, if the same genes control the clock and phenology in these populations, we expected variation in circadian period to be associated with phenological traits.

## Materials and Methods

### Sampling and Environmental Variation in the Sampled Area

**Figure 1.**
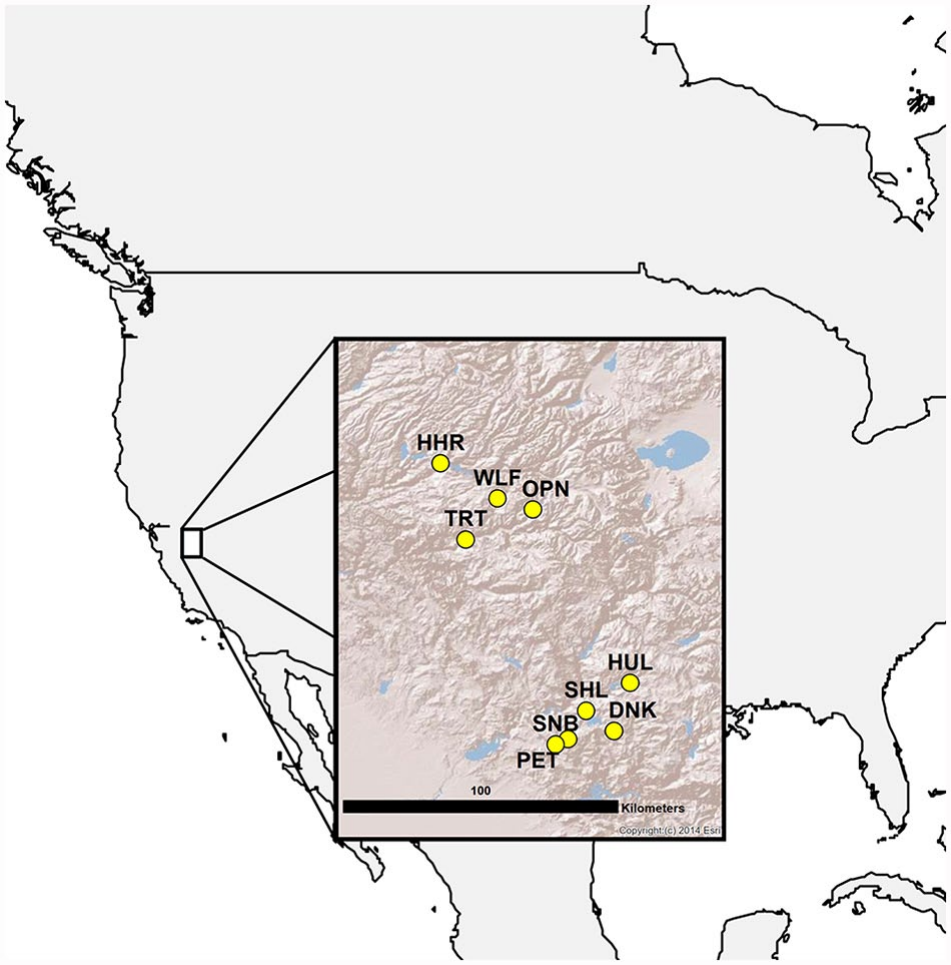
Sampling locations of *Mimulus laciniatus* populations in California. Insert map copyright © 2014 Esri.

We collected seeds from 9 populations of *M. laciniatus* at different elevations (~1000-2600 m above sea level) within an approximately 100-km radius in the Sierra Nevada Mountains in California, United States (Table 1; Fig. 1). Seeds in our experiment were either field collected (photoperiod experiment) or self-fertilized over 2 or 3 generations in the greenhouse (circadian period estimation). We characterized native environments of the sampled populations using data for 19 bioclimatic variables (30-s resolution, ~1- × 1-km grids) in the WorldClim data set (Fick and Hijmans, 2017). Molecular marker data suggest that genetic variation in this species in the area is largely among populations rather than among discrete elevational transects (Sexton et al., 2016); thus, we will consider our sample a single pool of populations.

**Table 1.** Sampling locations of *Mimulus laciniatus* populations in California and some of their environmental characteristics based on the WorldClim data.

Population	Latitude	Longitude	Elevation (m.a.s.l.)	AMT (°C)	TAR (°C)	AP (mm)
OPN	37°81′09″	119°48′52″	2590	3.8	31.9	726
WLF^a^	37°50′49″	119°35′63″	2395	4.9	30.4	777
HUL^b^	37°14′37″	119°09′61″	2171	7.0	30.2	868
DNK	37°05′11″	119°13′10″	1852	9.4	28.4	968
SHL	37°08′68″	119°18′39″	1594	11.0	30.0	893
TRT	37°42′99″	119°42′32″	1481	10.5	30.5	948
PET	37°03′35″	119°22′12″	1257	12.3	30.6	920
HHR	37°57′43″	119°47′16″	1208	10.8	31.4	904
SNB	37°02′32″	119°24′38″	1003	13.8	31.7	900

AMT = annual mean temperature; TAR = temperature annual range; AP = annual precipitation.

a. Included only in the photoperiod experiment

b. Included only in the circadian period estimation.

### Circadian Period Estimation

We used seeds from 8 populations of *M. laciniatus* for circadian period estimation, each represented by 6 families and 8 to 29 replicates per family. Seeds were stratified at +4 °C for 10 days before entrainment in 12-h/12-h light-dark cycles at +21 °C for 12 days. Plants were transferred into constant light and +21 °C 24 h prior to the start of imaging. All families were randomized and imaged for 5 days. We used the standard 12:12 h entrainment photoperiod, which represents different times of the season for the populations. We chose to use these conditions because longer day length during entrainment would have initiated elongation of shoots and development of flower buds, interfering with the leaf movement analysis. The temperature matches that used in the photoperiod experiment, making the results comparable. We estimated the length of the circadian period using the imaging-based leaf movement assay TRiP by Greenham et al. (2015). Following the same criteria used by Greenham et al. (2015), Lou et al. (2011), and Salmela et al. (2016), 8 individuals (out of 872) from 5 populations with circadian periods shorter than 18 h were discarded from the data.

### Estimation of Photoperiodic Responses and Phenology

We used 8 populations and 6 families per population to determine population differentiation in flowering responses and phenology under different photoperiods. We sowed several seeds per family in 2-inch pots with moist Fafard 4P mix. Pots with seeds from different families and populations were randomized on 5 sets of 6 trays. The seeds were first stratified in the dark at +4 °C for 10 days and assigned to the following treatments with light cycles (day length) of 11 h, 11 h 45 min, 12 h 30 min, 13 h 15 min, and 14 h (Environmental Growth Chambers, Chagrin Falls, Ohio, USA) at Duke University Phytotron. The range of these light-dark cycles corresponds to natural day lengths between mid-February and mid-May at a latitude of 37°, the populations’ origin. All chambers had a constant temperature of +21 °C and light intensity (PAR) of 700 µmol/m^−2^/s^−1^. We watered the plants daily, with no added fertilizer used. To minimize unwanted environmental effects within a chamber, locations of the pots in each tray and the positions of the trays in the chambers were re-randomized several times during the experiment. Slightly uneven germination of seeds from different populations and families resulted in a different number of seedlings in some populations in the photoperiod experiment (Suppl. Table S1) and some families not being represented in all day-length treatments (Suppl. Table S2).

We recorded the date of cotyledon emergence for the first germinating seed in each pot and transplanted extra seedlings from the same family or population in pots that had no successful germination in the same chamber. We monitored the plants daily for 14 weeks for the date of first open flower and the date of first ripe seed, when the first fully dried seed pod started dispersing seed. Days to cotyledon emergence was calculated as the number of days from the pots being moved to the growth chamber treatments after cold stratification. Flowering time was determined as the number of days from cotyledon emergence to the date of first open flower and seed-ripening time as the number of days from the first open flower to the date of the first ripe seed pod.

### Statistical Analyses

We used principal component analysis based on a correlation matrix to summarize variation in the 19 bioclimatic variables from the WorldClim data set. Components with eigenvalues greater than 1 were retained.

We analyzed variation in circadian period using analysis of variance (ANOVA). Population and family within population were considered random factors, such that their variance components and relative contributions to total phenotypic variation could be estimated using restricted maximum likelihood. We applied ANOVA to analyze variation also within each individual population, with only family included as a factor. To determine whether day length and population affected the propensity to flower in the photoperiod experiment, we used a generalized linear model with a binomial distribution and a logit link function. Genetic variation in days to cotyledon emergence, flowering time, and days to seed ripening was analyzed in the 14-h treatment only where 82 plants out of 91 flowered, with population as a factor in ANOVA. Family was not used as a factor in the models for traits other than circadian period because of the low replication within individual photoperiod treatments. We carried out all of the aforementioned analyses using IBM SPSS Statistics 25.

We used dose-response analysis in the package *drc* (Ritz et al., 2015) to estimate population-specific critical day lengths (50% of plants flowering) in R 3.5.1 (R Core Team, 2018). Estimates were obtained by fitting a 2-parameter log-logistic model on the proportion of plants of each seed family flowering in each day-length “dose” in the different treatments. Likelihood ratio tests were used to test for population differences in critical day-length estimates modeling flowering responses to photoperiod.

To describe potential clinal variation in traits, we tested for Pearson’s correlations between the populations’ home site conditions (elevation and PC2 based on the WorldClim data; we did not use PC1 because of its strong correlation with elevation) and trait averages in R. We applied the same approach to measure covariation among population averages of circadian period, critical day length, and flowering time.

## Results

### Environmental Variation in the Sampled Region

**Figure 2.**
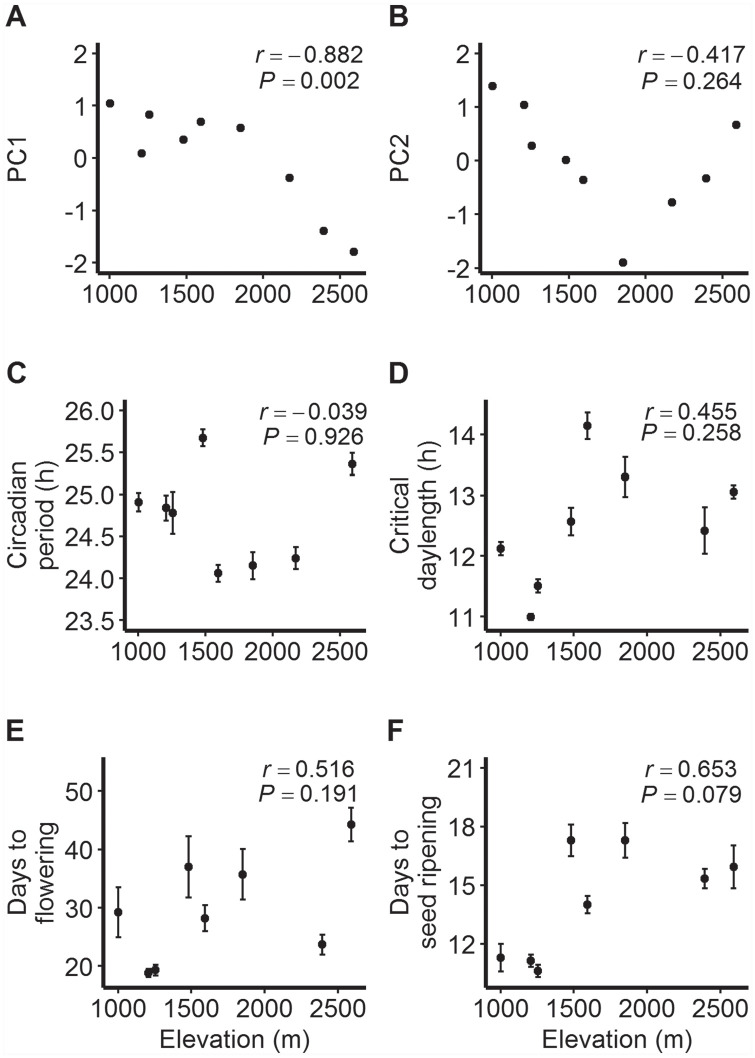
Relationships between elevation and 2 principal components, (a) PC1 and (b) PC2, that reflect home site climate conditions of the sampled populations, and between elevation and population means ±1SE (standard error) for (c) circadian period, (d) critical day length for flowering, (e) number of days to flowering from cotyledon emergence (14-h treatment), and (f) number of days to seed ripening from flowering (14-h treatment) for *Mimulus laciniatus* populations. Pearson’s correlation coefficients (*r*) and corresponding *p* values are shown.

WorldClim data indicated marked heterogeneity in temperature and precipitation conditions within the sampled region. For example, estimates of annual mean temperature varied from 3.8 °C at OPN to 13.8 °C at SNB, and estimates of annual precipitation ranged from 726 mm at OPN to 968 mm at DNK (Table 1). Principal component analysis identified 2 sets of correlated variables (Table 2), with the first component reflecting variation in overall temperature and rainfall (75.3% of total variation) and the second component accounting for the range of variation in temperature (17.4% of total variation). PC1 was significantly negatively correlated with elevation (*r* = −0.882, *p* < 0.01; Fig. 2a), reflecting lower temperature and precipitation at higher elevations. No significant linear correlation with elevation was found for PC2 (*r* = −0.417, *p* > 0.05; Fig. 2b), which was best associated with temperature annual range. Instead, PC2 exhibited a nonlinear trend: values first decreased between elevations 1003 m to 1852 m, after which they increased toward 2590 m. This signals the largest ranges of temperature at both low and high elevations.

**Table 2.** Summary of a principal component analysis on 19 climate variables in the WorldClim data set.^a^

	PC1	PC2
Bioclimatic variable	14.3 (75.3%)	3.3 (17.4%)
Annual mean temperature	0.958	0.277
Mean diurnal range	−0.587	0.719
Isothermality	−0.725	−0.0620
Temperature seasonality	0.241	0.929
Maximum temperature of warmest month	0.891	0.444
Minimum temperature of coldest month	0.986	0.125
Temperature annual range	−0.283	0.946
Mean temperature of wettest quarter	0.973	0.214
Mean temperature of driest quarter	0.927	0.363
Mean temperature of warmest quarter	0.940	0.337
Mean temperature of coldest quarter	0.979	0.188
Annual precipitation	0.895	−0.264
Precipitation of wettest month	0.935	−0.270
Precipitation of driest month	−0.957	0.108
Precipitation seasonality (coefficient of variation)	0.949	−0.141
Precipitation of wettest quarter	0.946	−0.270
Precipitation of driest quarter	−0.899	0.160
Precipitation of warmest quarter	−0.977	−0.0510
Precipitation of coldest quarter	0.904	−0.369

a. Values in the table mark loadings (Pearson’s correlation coefficients) of the bioclimatic variables on 2 principal components. Strong correlations are shaded in gray. Eigenvalues for both components are shown, with values in parentheses indicating how much of total variation each component explained.

### Natural Variation in Circadian Period Length

The circadian clock was genetically variable at 2 sampling levels. The length of the free-running circadian period varied significantly among populations (Table 3), and these patterns yielded a larger variance component for population (0.301, 12.4% of total variation) than for family within population (0.0509, 2.1% of total variation) in the full data set. Mean period length ranged from 24.1 h in the SHL population to 25.7 h in the TRT population (Fig. 2c). Overall, there was moderate evidence for variation among seed families within populations (Table 3). When tested separately within each population, the HHR and SHL populations showed significant among-family differentiation (Suppl. Table S3). Family means for circadian period length varied between 23.9 and 25.7 h in the HHR population and between 23.4 and 24.8 h in the SHL population (Suppl. Table S4).

**Table 3.** Analysis of variance for natural variation in circadian period and for timing of phenological transitions in the 14-h day-length treatment.

Trait	*df*	Mean square	*F*	*p*
Circadian period				
Population	7	31.487	10.514	<0.0001
Family (within population)	40	3.048	1.467	0.033
Residuals	816	2.078		
Days to cotyledon emergence (14-h treatment)				
Population	7	13.917	1.390	0.223
Residuals	74	10.015		
Days to flowering from cotyledon emergence (14-h treatment)				
Population	7	1010.375	9.035	<0.0001
Residuals	74	111.830		
Days to seed ripening from flowering start (14-h treatment)				
Population	7	78.221	13.346	<0.0001
Residuals	71	5.861		

Among-population differentiation in flowering responses to photoperiod and timing of phenological events.Across photoperiod treatments, longer day lengths increased the flowering propensity (Wald chi-square = 75.4, *df* = 4, *p* < 0.0001). Further, we observed population differentiation in flowering propensity (Wald chi-square = 80.5, *df* = 7, *p* < 0.0001; Table 4), indicating that some populations required longer photoperiods for flowering. Critical day-length estimates for flowering (defined as 50% of plants flowering in each population based on dose-response analysis) varied significantly among populations (likelihood ratio value = 255.35, *df* = 7, *p* < 0.0001; Fig. 2d), ranging from 11.0 h in the HHR population to 14.1 h in the SHL population (Table 4; Suppl. Figure S1).

**Table 4.** Proportion of *Mimulus laciniatus* plants from each population that flowered in different photoperiod treatments.^a^

Population	Day-Length Treatment					CDL Estimate	
	11 h	11 h 45 min	12 h 30 min	13 h 15 min	14 h	Mean ± SE (h)	Corresponding Date
OPN	0.00	0.00	0.06	0.75	0.94	13.06 ± 0.11	14 April
WLF	0.00	0.00	0.80	1.00	1.00	12.42 ± 0.38	28 March
DNK	0.00	0.00	0.00	0.29	1.00	13.30 ± 0.33	20 April
SHL	0.00	0.00	0.00	0.07	0.38	14.14 ± 0.22	16 May
TRT	0.00	0.00	0.25	1.00	1.00	12.57 ± 0.23	1 April
PET	0.12	0.69	1.00	1.00	1.00	11.51 ± 0.11	6 March
HHR	0.53	1.00	1.00	1.00	1.00	10.99 ± 0.05	20 February
SNB	0.00	0.19	0.80	1.00	1.00	12.12 ± 0.11	21 March

a. Estimates of critical day length for flowering (CDL) based on dose-response analysis are shown (mean ± SE), along with roughly estimated corresponding dates when the populations encounter these day lengths in the wild. Populations ordered by decreasing elevation from top to bottom.

Days to flowering (from cotyledon emergence) and days to seed ripening (from flowering start) in the 14-h day-length treatment were significantly different among populations (Table 3). In general, flowering started more rapidly in long days than in short days, whereas photoperiod length in general did not seem to affect days to cotyledon emergence or seed ripening (Suppl. Fig. S2). The HHR and PET populations were fastest to start flowering (mean 18.8 and 19.3 days, respectively), whereas the OPN population started flowering the latest (mean 44.3 days; Fig. 2e). Seed ripening was fastest in the PET, HHR, and SNB populations (mean 10.6, 11.1, and 11.3 days, respectively) and slowest in the TRT and DNK populations (mean 17.3 days; Fig. 2f).

### Correlations between Environmental Variables and Phenotypic Traits

**Figure 3.**
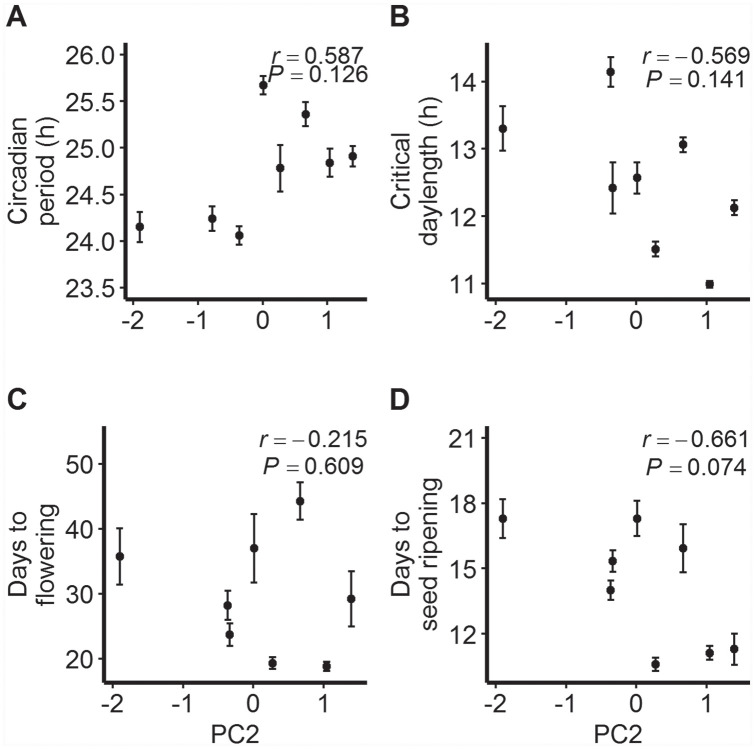
Relationships between PC2 (a composite estimate of on-site temperature variability) and population means ±1SE (standard error) for (a) circadian period, (b) critical day length for flowering, (c) number of days to flowering from cotyledon emergence (14-h treatment), and (d) number of days to seed ripening from flowering (14-h treatment) for *Mimulus laciniatus* populations. Pearson’s correlation coefficients (*r*) and corresponding *p* values are shown.

None of the tested linear relationships between elevation and population means for the studied traits were statistically significant (Fig. 2c-f). However, scatterplots for circadian period and critical day length suggested nonlinear trends with elevation. The highest-elevation population OPN at 2590 m had a circadian period length similar to that observed in TRT at 1481 m; the trait average was lowest in mid-elevation populations (Fig. 2c). Further, mid-elevation populations required longer photoperiods for flowering than those located below 1500 m, but the highest-elevation populations had a shorter critical day length than those at mid elevations (Fig. 2d). Regressions with quadratic terms were not statistically significant (*p* > 0.250). Associations between the 2 traits and PC2 were stronger and moderate (positive for circadian period, negative for critical day length) but statistically nonsignificant (Fig. 3a, b). The correlation between elevation and flowering time in a 14-h photoperiod was positive and moderate (Fig. 2e) but statistically nonsignificant; in addition, the negative correlation with PC2 was nonsignificant (Fig. 3c). The strongest evidence of a correlation, albeit nonsignificant at α = 0.05, was that between PC2 and days to seed ripening (*r* = −0.661, *p* = 0.074; Fig. 3d).

### Associations between Traits

No statistically significant correlations were found between population means of circadian period and critical day length or phenological traits (Suppl. Fig. S3). Critical day-length estimates and phenological traits showed a trend of positive correlation, but none of the comparisons were statistically significant (Suppl. Fig. S4).

## Discussion

Mountainous areas provide a fascinating foundation for exploring adaptation in relation to consequential small-scale environmental heterogeneity across elevational gradients. In our study, we found differentiation among populations in various types of biological rhythms in the annual plant *M. laciniatus* sampled at 1003 to 2590 m in the Sierra Nevada Mountains in California. The circadian clock and timing of reproduction were variable among populations, but despite marked climatic heterogeneity within the region, we found no statistical support for linear elevational clines or for strong trait covariation.

### Circadian Rhythms Vary among Populations from Different Elevations

The plant circadian clock is considered to facilitate adaptation to predictable day-night cycling, maintaining rhythms approximately 24 h in length in a suite of functional traits (Greenham and McClung, 2015; Millar, 2016). Here, we found significant variation in the length of the free-running circadian period among plant populations from different elevations. The range of differentiation in period among populations (~1.5 h) in the Sierra Nevada was smaller than that in an assorted global selection of 150 *A. thaliana* genotypes (Michael et al., 2003), but as a result of our comprehensive replication within populations, the observed differences were statistically highly significant. Further, the range was slightly larger than that among 4 populations of *B. stricta* at 2500 to 3000 m in southeastern Wyoming (Salmela et al., 2016). Our sampling protocol was similar to that in Salmela et al. (2016), permitting measurements of genetic diversity also among seed families within populations. At this level, we observed genetic variation within 2 populations only, with similar ranges of diversity to that seen among populations. The amount of diversity on this local scope is smaller than in annual populations of *M. guttatus* along the western coast of North America and at elevations below 650 m (Greenham et al., 2017) or within 2 populations of *B. stricta* at 2500 m in the central Rocky Mountains with circadian period ranges up to 3.5 h (Salmela et al., 2016). Differences between the studies may arise from variable levels of overall within-species genetic diversity, from variation in sampling strategies, or from species differences in life history (annual vs. perennial). Overall, causes of within-population variation in the clock have yet to be identified, but they could be related to spatially or temporally varying selection on closely related traits such as flowering time (Exposito-Alonso et al., 2018; Troth et al., 2018).

Photoperiodic variation is considered central in driving large-scale natural variation in the circadian clock (Hut et al., 2013), but we still do not know which particular environmental factors affect the distribution of genetic diversity in the circadian clock (Salmela and Weinig, 2019). In the current study, which used a narrow latitudinal range (cf. Edwards et al., 2005), the circadian period varied among populations from differing elevations, but we did not detect evidence for a straightforward clinal pattern amidst this diversity. Period length was long at both low and high elevations, with shorter population averages at mid elevation. This suggests environmental factors tightly associated with PC1, namely, average temperature and precipitation, are not primary drivers of clock variation in this region. Instead, the modest but statistically nonsignificant positive correlation between PC2 and period points in the direction of circadian period being associated with the variability of local environments, with longer periods observed at sites with wider estimated diurnal or seasonal ranges in temperature. It is important to consider that actual environmental conditions at the sampled sites may differ from the model-based WorldClim estimates (with a 1- × 1-km grid) due to the complex regional topography. Also, among-year variation in temperature and precipitation is likely to be substantial, an element not reflected in estimates of long-term averages by WorldClim but evident in recorded data from weather stations located in the area (California Data Exchange Center, https://cdec.water.gov/). Statistically sounder descriptions of nonlinear clines demand more intensive sampling of populations across the elevational gradient.

### Differentiation in Flowering Responses to Photoperiod and in Phenological Traits

Genetic diversity in the clock is abundant in natural populations, but its patterns have rarely been examined jointly with other important and genetically related adaptive traits such as photoperiodic responses (Salmela and Weinig, 2019). In our sample of populations that vary in circadian period, we found pronounced differentiation in the critical day-length requirement for flowering—a potential adaptation to habitats with a different timing of the growing season. The population range of critical day length was from 11 to 14 h. This photoperiod requirement in our study system is very strict, as the plants kept developing only leaves and vegetative branches when grown under the critical day-length threshold (data not shown). At 37°, where our sampling was conducted, a day length of 11 h is reached in mid-February and 14 h in mid-May.

Paralleling our observations for circadian period, the spatial patterns of population variation along the elevational gradient were more obscure than hypothesized. As expected, the lowest-elevation populations flowered already in shorter photoperiods, but the maximum critical day length occurred at mid elevation rather than in the highest-elevation populations at 2500 m. Thus, our results are not compatible with a simple linear association between elevation and critical day length along the sampled transect. This outcome differs from those on the closely related *M. guttatus*, in which plants from lower elevations sampled across multiple elevational gradients in the Sierra Nevada and the Cascades Mountains had a shorter critical day-length requirement than plants from higher elevations sampled at ~1500 m (Kooyers et al., 2015). An analogous pattern was evident in the study by Friedman and Willis (2013), which included 46 populations sampled between California and Alaska. In the current study, an estimate of daily and seasonal variability within each site (PC2) was more strongly associated with critical day length for flowering than was elevation, with longer day lengths at sites with less temperature variability. We speculate that the absence of a linear cline in *M. laciniatus* could be explained by the relatively late onset of optimal growing conditions at high elevations, where the growing season is likely to be limited by snow cover and low temperatures and where the critical photoperiod requirement needs to be exceeded before shortening day lengths after the summer solstice. Photoperiod is thought to be a stronger signal of the beginning of the growing season than temperature at low compared with high elevations, as was suggested by a study of *A. thaliana*, in which plants from lower elevations showed stronger photoperiodic responses than plants from higher elevations (Lewandowska-Sabat et al., 2017). Elevational gradients can be confounded by other factors such as direction of exposure (e.g., south vs. north) and steepness of the slope that can affect daily ground temperature regimes and moisture levels at the ground level. Hence, detailed knowledge of such site-specific microenvironmental variation, estimated, for instance, with data loggers over multiple years, can yield sharper clinal patterns in adaptive traits than the use of rougher-scale spatial or climatic proxies such as elevation (Lampei et al., 2019).

The correct timing of flowering is considered crucial to fitness in plants, and appropriately, the trait is among the most intensively investigated ones in studies on local adaptation. In our study, we observed marked differentiation among populations in flowering time (calculated from cotyledon emergence) in the treatment that had a photoperiod long enough to fulfill the critical day-length requirement of 7 out of the 8 populations. Long days accelerated flowering in our photoperiod experiment, as has been found in other studies on long-day plants such as *A. thaliana* and other *Mimulus* spp. (Giakountis et al., 2010; Friedman and Willis, 2013). These differences could arise due to endogenous signals determining the length of the juvenile period, or they could be due to differences in developmental rates among populations (Amasino, 2010). We also found a difference among populations in days to seed ripening after flowering start, suggesting that the development of both flowers and ripe seeds occurs at a different rate in different populations. In natural conditions, both need to occur before the growing season ends. *M. laciniatus* grows on moss patches on granite outcrops that dry out during the summer, thus requiring that seeds have developed by that time. Subsequently, the end of the growing season could be a strong selective force. Delayed flowering does provide the possibility of growing more leaves before entering a reproductive phase, which increases the potential resources for seed production.

As in circadian period and critical day length, we did not observe clear-cut clinal variation in flowering time. The trend between elevation and population means was positive, suggesting slower development at higher elevations, but this was not statistically significant. Still, its direction agrees with the results by Dickman et al. (2019), who surveyed flowering time in a set of 9 *M. laciniatus* populations in a greenhouse environment. Kooyers et al. (2015) documented notable genetic diversity in flowering time among 52 annual populations of *M. guttatus*, but a significant latitude × elevation interaction signaled that the trait’s environmental associations vary depending on the exact spatial locations of populations. On the other hand, in *A. thaliana* sampled across 7° of latitude in the Iberian Peninsula, elevation correlated positively with flowering time measured in a 16-h photoperiod, explaining 38% of the variation among 300 genotypes (Vidigal et al., 2016). As compared with these wide-ranging studies, sampling here was more spatially focused, but nonetheless, it is important to note that in natural settings, a 14-h photoperiod used here might coincide with nonoptimal reproductive conditions for some of the lower-elevation populations that initiate flowers early in spring and thus in shorter day length. Moreover, it is possible that population variation in response to photoperiod and in flowering time within a given photoperiod are indicative of adaptation to divergent environmental factors (Kooyers et al., 2015).

### Relationships between Traits That Measure Time

The common genetic bases of the circadian clock and phenological responses suggest phenotypic covariation between traits that measure time at different scales (e.g., Brachi et al., 2010; Lou et al., 2011; Suárez-López et al., 2001). We documented population-level variation in the clock and in timing of reproduction, but we found no statistically significant correlations between population trait means. In contrast, in a within-population sample in *B. stricta* with more genetic diversity in circadian period (~3.5 h), maternal families with longer period lengths in leaf movement tended to flower earlier and at a larger size (Salmela et al., 2018). Here, the correlation between circadian period and critical day length was similar in direction but nonsignificant with only 8 populations. Although it is possible based on our data that the circadian rhythm in leaf movement and phenology are not interconnected in this species, differences between study systems and experimental designs may also contribute to these patterns. We recorded circadian period in a 12-h photoperiod, whereas critical day length for flowering was estimated across a range of ecologically relevant photoperiods. Naturally variable conditions can influence the estimates of circadian parameters in a genotype-specific manner (e.g., Rubin et al., 2017). Also, Salmela et al. (2018) applied a more natural, hourly changing temperature cycle that was used in both the circadian and flowering time assay and that was based on weather station data near the study population’s home site; in the current study, traits were scored in 21 °C. To understand the interplay between natural clock variation and adaptation in natural settings, it will be important to conduct thorough phenotypic assessments of both circadian rhythms and related functional traits in similar environmental conditions. Furthermore, because an overlap between groups of genes that affect 2 distinct traits does not necessarily translate into a strong correlation between phenotypes, a larger and more diverse genotypic sample across varying geographic distances will be valuable in defining the expected clock-phenology relationship more precisely.

## Conclusions

In this experiment, we documented naturally occurring genetic variation in circadian period length and flowering responses to photoperiod among plant populations from the Sierra Nevada. The sampled locality is highly variable in terms of temperature and precipitation depending on elevation and fine-scale topography, yet we did not find well-defined clinal diversity in biological rhythms. This outcome may partially arise from challenges in accurately estimating native environmental conditions of the populations in complex terrains. Further, although it has been shown that the same genes control variation in the circadian clock and flowering responses, for instance, in *A. thaliana*, we did not detect significant correlations between these traits. Future studies should focus on examining the ecological significance of genetic variation in circadian period in natural populations (both among and within) and further examine the possible connection between the circadian clock and phenological traits that are more intensively studied in the context of local adaptation and whose association to fitness is thus better characterized.

## Supplemental Material

Supplementary_revised – Supplemental material for Populations Are Differentiated in Biological Rhythms without Explicit Elevational Clines in the Plant Mimulus laciniatus Click here for additional data file. Supplemental material, Supplementary_revised for Populations Are Differentiated in Biological Rhythms without Explicit Elevational Clines in the Plant Mimulus laciniatus by Päivi H. Leinonen, Matti J. Salmela, Kathleen Greenham, C. Robertson McClung and John H. Willis in Journal of Biological Rhythms
