# Supplementary material for: Populations Are Differentiated in Biological Rhythms without Explicit Elevational Clines in the Plant Mimulus laciniatus
Source: J Biol Rhythms. 2020 Jul 6;35(5):452–64. doi: 10.1177/0748730420936408 (PMC7534027; doi:10.1177/0748730420936408)
Supplement: Supplementary_revised – Supplemental material for Populations Are Differentiated in Biological Rhythms without Explicit Elevational Clines in the Plant Mimulus laciniatus [file Supplementary_revised.pdf]

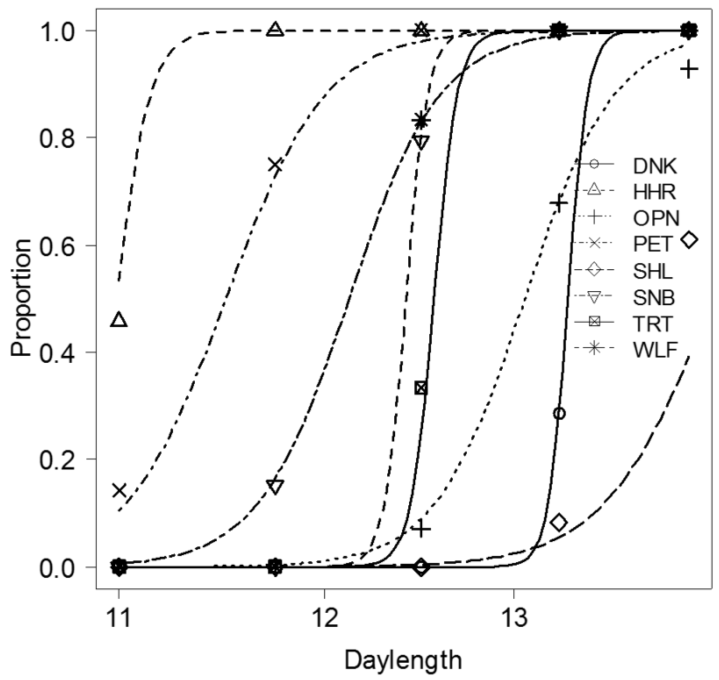

Supplementary figure S1. Fitted dose-response curves of critical photoperiod for flowering for *Mimulus laciniatus* populations originating from different elevations.

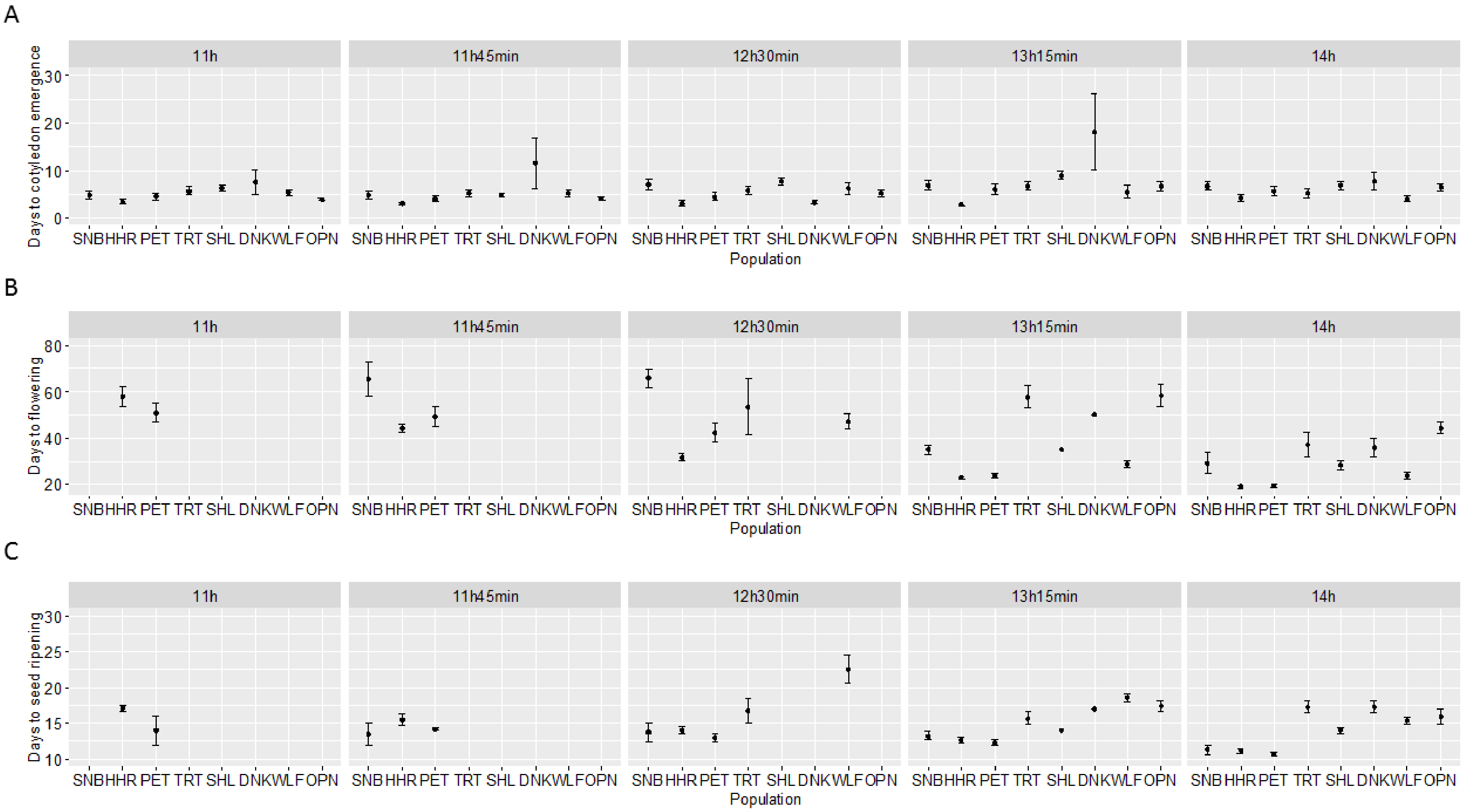

Supplementary figure S2. Mean ±1SE (standard error) for number of days to cotyledon emergence, days to flowering and days to seed ripening for *Mimulus laciniatus* populations originating from different elevations under different daylength treatments.

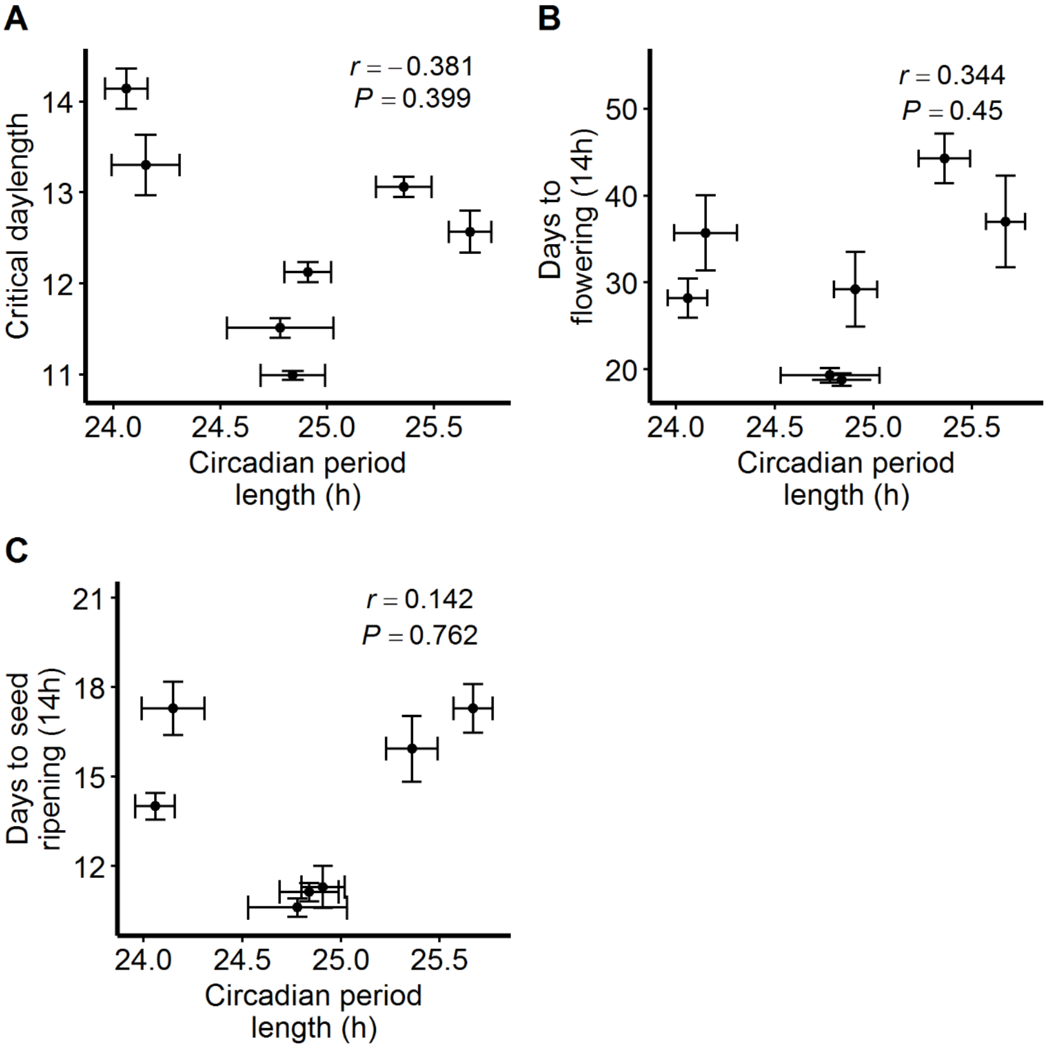

Supplementary figure S3. Relationships between populations means for circadian period ±1SE (standard error) and a) critical daylength for flowering, mean ±1SE (b) number of days to flowering from cotyledon emergence (14-h treatment) and (c) number of days to seed ripening from flowering (14-h treatment) for *Mimulus laciniatus* populations. Pearson's correlation coefficients (*r*) and corresponding *P*-values are shown.

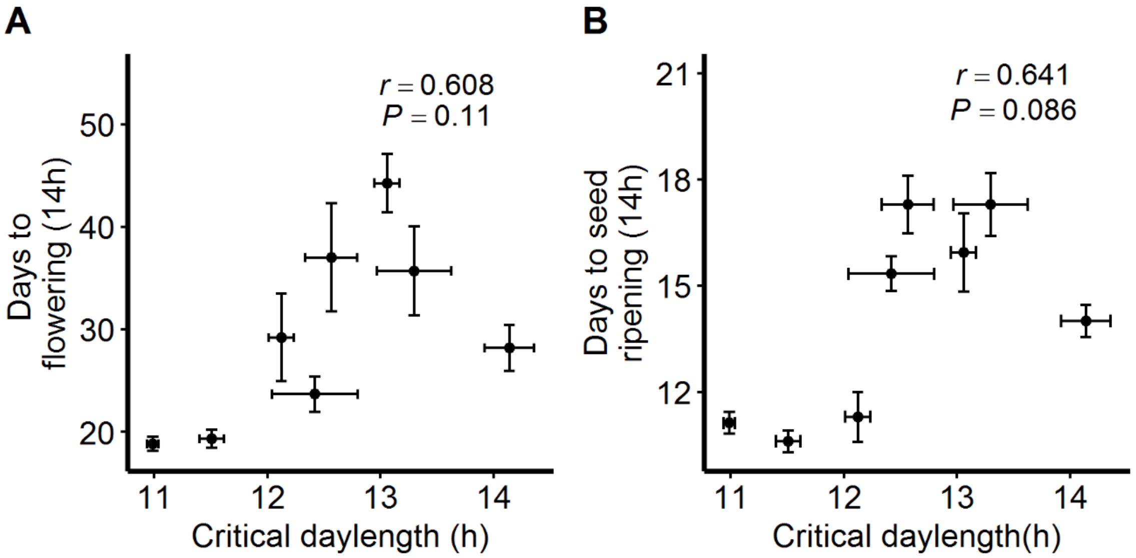

Supplementary figure S4. Relationships between critical daylength estimates ±1SE and mean ±1SE a) number of days to flowering from cotyledon emergence (14-h treatment) and (b) number of days to seed ripening from flowering (14-h treatment) for *Mimulus laciniatus* populations. Pearson's correlation coefficients (*r*) and corresponding *P*-values are shown.

Supplementary table S1. Number of plants from each population and (number of families in brackets) in each photoperiod treatment and in the circadian period experiment. Populations are ordered by decreasing elevation from top to bottom.

Population	Daylength treatment					Circadian period
	11H00	11H45	12H30	13H15	14H00	
OPN	16 (8)	16 (8)	16 (7)	16 (7)	16 (7)	107 (6)
WLF	14 (7)	15 (7)	10 (4)	11 (6)	6 (4)	.
HUL	.	.	.	.	.	115 (6)
DNK	12 (6)	6 (2)	3 (3)	7 (7)	7 (5)	106 (6)
SHL	15 (6)	16 (8)	16 (7)	15 (4)	13 (6)	97 (6)
TRT	14 (5)	16 (6)	16 (7)	16 (6)	8 (4)	112 (6)
PET	16 (7)	16 (8)	15 (6)	16 (6)	10 (5)	76 (6)
HHR	15 (8)	16 (8)	16 (8)	16 (8)	16 (8)	103 (6)
SNB	16 (6)	16 (6)	15 (3)	16 (4)	15 (4)	148 (6)

Supplementary table S2. Number of plants from each family in each photoperiod treatment and circadian period estimation. Populations are ordered by decreasing elevation from top to bottom.

Family	Treatment					Circadian period
	11H00	11H45	12H30	13H15	14H00	
OPN-1	2	3	4	4	3	18
OPN-2	2	2	2	2	1	21
OPN-3	2	1	0	0	2	.
OPN-4	2	2	2	2	3	.
OPN-5	2	2	2	2	0	22
OPN-6	2	2	2	2	3	13
OPN-7	2	2	3	1	2	19
OPN-8	2	2	1	3	2	.
OPN-10	.	.	.	.	.	14
WLF-63	2	5	3	4	2	.
WLF-64	2	2	5	1	1	.
WLF-65	1	1	1	2	0	.
WLF-66	1	2	1	0	0	.
WLF-74	4	2	0	0	2	.
WLF-75	2	0	0	1	0	.
WLF-76	2	2	0	1	1	.
WLF-77	0	1	0	2	0	.
HUL2	.	.	.	.	.	11
HUL6	.	.	.	.	.	21
HUL9	.	.	.	.	.	25
HUL10	.	.	.	.	.	28
HUL12	.	.	.	.	.	14
HUL19	.	.	.	.	.	16
DNK-9	1	0	0	1	0	17
DNK-10	2	0	0	1	0	26
DNK-11	1	0	0	0	2	.
DNK-12	0	0	1	1	0	.
DNK-13	2	0	1	1	1	11
DNK-14	2	2	0	1	2	15
DNK-16	0	0	0	1	1	15
DNK-17	4	4	1	1	1	22
SHL-3	0	2	1	2	0	.
SHL-6	3	2	1	0	1	.
SHL-16	2	2	4	1	1	19
SHL-17	2	2	1	0	6	17
SHL-19	1	2	0	0	0	.

SHL-21	.	.	.	.	.	18
SHL-22	4	2	2	3	1	22
SHL-23	0	2	5	9	3	13
SHL-24	3	2	2	0	1	8
TRT-3	2	4	5	2	1	.
TRT-5	0	0	2	1	0	13
TRT-6	1	3	1	0	0	15
TRT-12	3	2	1	4	4	20
TRT-13	0	1	1	1	0	21
TRT-14	4	3	3	2	2	26
TRT-15	4	3	3	6	1	17
PET-2	5	2	3	5	5	16
PET-6	1	2	2	3	1	11
PET-7	2	2	2	0	0	16
PET-10	2	2	4	2	1	8
PET-16	2	3	0	1	0	.
PET-17	2	3	1	4	2	11
PET-18	0	1	0	0	1	.
PET-20	2	1	3	1	0	14
HHR-1	2	2	3	2	3	12
HHR-6	2	2	1	2	1	21
HHR-7	3	2	2	2	1	19
HHR-9	1	2	2	2	2	19
HHR-10	2	2	2	2	3	12
HHR-12	2	2	2	2	2	20
HHR-14	2	2	2	2	2	.
HHR-17	1	2	2	2	2	.
SNB-1	2	3	0	4	0	29
SNB-2	.	.	.	.	.	26
SNB-4	2	2	6	4	4	27
SNB-7	2	3	5	3	1	19
SNB-11	2	2	0	0	0	.
SNB-12	2	2	4	5	4	21
SNB-15	6	4	0	0	6	26

Supplementary table S3. Results of one-way ANOVA for among-family differences for each population in circadian period. Populations are ordered by decreasing elevation from top to bottom. The two populations with significant family variation are shown in bold and italics.

Population	df	MS	*F*	*P*
OPN	5	1.185	0.601	0.699
HUL	5	1.721	0.830	0.531
DNK	5	3.160	1.193	0.318
SHL	***5***	***2.941***	***3.480***	***0.006***
TRT	5	1.743	1.512	0.192
PET	5	4.385	0.895	0.489
HHR	***5***	***6.414***	***2.967***	***0.016***
SNB	5	2.837	1.587	0.167

Supplementary table S4. Family means in circadian period length for *Mimulus laciniatus* populations. The two populations with significant among-family variation are marked in bold and italics.

Population	Family	Period mean (h)
OPN	OPN1	25.6
OPN	OPN2	25.5
OPN	OPN5	25.2
OPN	OPN6	25.4
OPN	OPN7	25.5
OPN	OPN10	24.9
HUL	HUL2	24.5
HUL	HUL6	24.7
HUL	HUL9	24.2
HUL	HUL10	24.1

HUL	HUL12	24.0
HUL	HUL19	24.0
DNK	DNK10	24.3
DNK	DNK11	24.0
DNK	DNK13	24.3
DNK	DNK14	23.5
DNK	DNK16	24.9
DNK	DNK17	24.1
SHL	***SHL16***	***24.0***
SHL	***SHL17***	***23.4***
SHL	***SHL21***	***24.3***
SHL	***SHL22***	***24.0***
SHL	***SHL23***	***24.3***
SHL	***SHL24***	***24.8***
TRT	TRT5	25.7
TRT	TRT6	26.2
TRT	TRT12	25.4
TRT	TRT13	25.7
TRT	TRT14	25.7
TRT	TRT15	25.3
PET	PET2	24.2
PET	PET6	25.0
PET	PET7	24.8
PET	PET10	25.3
PET	PET17	24.1
PET	PET20	25.5
HHR	***HHR1***	***23.9***
HHR	***HHR6***	***25.7***
HHR	***HHR7***	***24.8***
HHR	***HHR9***	***25.1***
HHR	***HHR10***	***24.8***
HHR	***HHR12***	***24.4***
SNB	SNB1	24.6
SNB	SNB2	25.0
SNB	SNB4	25.3
SNB	SNB7	24.9
SNB	SNB12	24.4
SNB	SNB15	25.1
